# Multiple periodic solutions of a delayed predator–prey model with non-monotonic functional response and stage structure

**DOI:** 10.1080/17513758.2014.920530

**Published:** 2014-05-23

**Authors:** Yingyuan Liu, Xiaolan Zhang, Tiejun Zhou

**Affiliations:** ^a^ College of Science, Hunan Agricultural University, Changsha, Hunan410128, People's Republic of China

**Keywords:** stage structure, non-monotone functional response, multiple periodic solutions, predator–prey system, 92B05, 34C25

## Abstract

The paper studies a periodic and delayed predator–prey system with non-monotonic functional responses and stage structure. In the system, both the predator and prey are divided into immature individuals and mature individuals by two fixed ages. It is assumed that the immature predators cannot attack preys, and the case of the mature predators attacking the immature preys is also ignored. Based on Mawhin's coincidence degree, sufficient conditions are obtained for the existence of two positive periodic solutions of the system. An example is presented to illustrate the feasibility of the main results.

## 1. Introduction

In a classical predator–prey model, it is generally assumed that there are no differences among the individuals of each species, which implies all the predators have the same survival probability and the same fertility, and all the preys also have the same survival probability and the same fertility. It is also assumed that each individual predator has the same attacking ability and each individual prey faces the same risk of being attacked. However, this phenomenon of no differences among individuals is very rare in the natural world. For example, the fertility and the attacking ability between an infant lion and an adult lion are apparently different. It is more reasonable to divide a species into different stages based on age. A simple method is to divide a species into two stages, the immature stage and the mature stage, where the immature individuals generally have no fertilities. There are different ways to impose the stage structure in the model, but usually only one species is taken into the consideration, for example, a stage structure for predator with fertility. Some systems consider stage structures only for the predator [2,11,14,15,17,25], and some consider stage structures only for the prey [8,9,19–22,28,29]. In fact, a more general and more realistic system considers a stage structure for both the predator and the prey [5,6,13,16,27]. In these systems with stage structures for both the predator and prey, immature predator attacking prey is generally ignored [13]. In addition, considering that the immature preys are usually protected by their parents, the probability of immature prey being attacked is very small and therefore mature predator attacking immature prey can also be ignored [13,27]. In [27], the following predator–prey model with stage structure for both the predator and prey is studied.

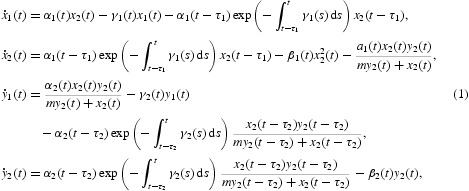

where *x*_1_(*t*) and *x*_2_(*t*) denote the densities of the immature and mature individual preys at time *t*, respectively; *y*_1_(*t*) and *y*_2_(*t*) represent the densities of the immature and mature individual predators at time *t*, respectively. The term 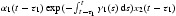 represents the number of immature preys that were born at time *t*−τ_1_, still survive at time *t*, and transfer from the immature stage to the mature stage at time *t*. The term

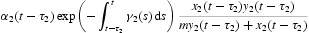

represents the number of immature predators that were born at time *t*−τ_2_, still survive at time *t*, and transfer from the immature stage to the mature stage at time *t*. It is assumed in Equation (1) that the immature predators do not feed on preys and the mature predators only feed on the mature preys. Sufficient conditions are given for the permanence and existence of a positive periodic solutions to model (1) in [27]. A stage-structured predator–prey system with functional response is an important population model, and it has been extensively studied recently. In these systems, three kinds of monotone functions *g*(*x*)=*mx, mx*/(*a*+*x*), 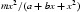, where *x* denotes the density of prey, are often used [2,8,9,11,13–15,17,19,21,29]. These functional response functions are monotonic for prey. But some experiments and observations indicate that a non-monotonic response also occurs under certain circumstances. For this reason, Andrews [1] suggested the following function to model the non-monotonic response:

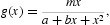

where *m, a*, and *b* are positive constants, which is called the Holling type-IV function. Its simplified form is 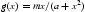. There are many researches on the predator–prey with non-monotonic response [18,23–26,28]. For example, Mischaikow and Wolkowicz [18], Wolkowicz [23] considered a general non-monotonic response function *p*(*x*), which was assumed to satisfy the following three conditions:

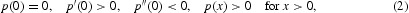

there exists *h*>0 such that

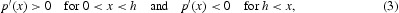

and

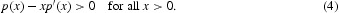

Obviously, the function *g*(*x*) above satisfies these assumptions. Xia *et al.* [26] also considered a general non-monotonic response function *p*(*x*), which was assumed to satisfy the following three conditions:

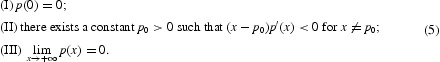

It is easy to see that the function *g*(*x*) above also satisfies the conditions (I)–(III).

Recently, some researchers incorporated the stage structure and the non-monotonic response into the predator–prey model [25,28]. For example, Yang *et al.* [28] considered the following predator–prey system with Holling type-IV functional response and stage structure for prey in a periodic environment:

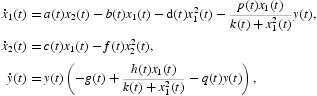

where *x*_1_(*t*) and *x*_2_(*t*) denote the density of the immature and mature prey, respectively, and *y*(*t*) is the density of predator that preys on *x*_1_. Xia *et al.* [25] considered the following predator–prey system with Holling type-IV functional response and stage structure for predator in a periodic environment:

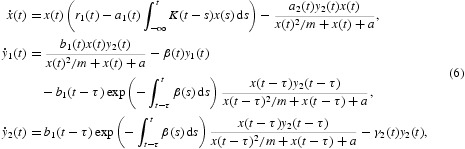

where *y*_1_(*t*) and *y*_2_(*t*) denote the density of the immature and mature predator, respectively, and *x*(*t*) is the density of prey.

Because of the periodicity of the environment, researchers not only care about the permanence and extinction of predator–prey systems, but also concern about the periodic change of these systems [13,25,27]. For example, by applying the method of coincidence degree, the authors of Xu *et al.* [27] studied the existence of a positive periodic solution to system (1). At the same time, in order to explain the diversity of some systems, the multistability or multiperiodicity of those system are considered [3,4,7,25]. For example, the authors of Xia *et al.* [25] obtained some sufficient conditions for the existence of at least two positive periodic solutions to system (6).

However, the combined effects of Holling type-IV functional response and the stage structure for both the predator and prey on a predator–prey model has not yet been widely studied. The motivation of this paper is to study the following delayed predator–prey system by replacing the ratio-dependent response function 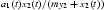 of system (1) with the Holling type-IV function 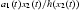 where 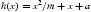.

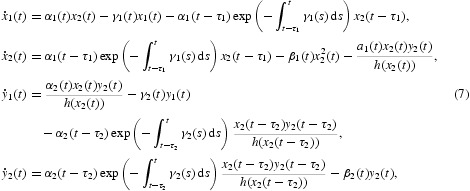

where α_1_(*t*), α_2_(*t*), β_1_(*t*), β_2_(*t*), γ_1_(*t*), γ_2_(*t*) and *a*_1_(*t*) are continuous positive periodic functions with period ω, the constants *m* and *a* are positive.

The initial conditions in Equation (7) are of the form

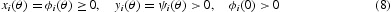

for *i*=1, 2, 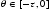, where 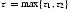, 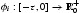, 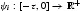 are continuous positive periodic functions. The symbol ℝ^+^ in the above denotes the set of all positive real numbers, and the symbol 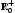 denotes the set of all the non-negative real numbers. The main purpose of this paper is to obtain some sufficient conditions for the existence of multiple positive periodic solutions to system (7).

## 2. Main results

In order to prove the existence of positive periodic solutions to system (7), we first summarize some relative concepts and results from [10] in the following.

Let *X* and *Z* be normed vector spaces, 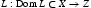 be a linear mapping, and 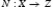 be a continuous mapping. The mapping *L* is called a Fredholm mapping of index zero if 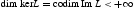 and Im *L* is closed in *Z*. If *L* is a Fredholm mapping of index zero then there exist continuous projectors 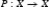, and 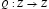 such that 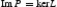, and 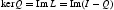. It follows that 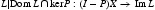 is invertible. We denote the inverse of the map by *K*_*p*_. If Ω is an bounded subset of *X*, the mapping *N* is then called *L*-compact on 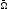 if 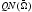 is bounded and 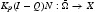 is compact. Since Im *Q* is isomorphic to ker *L*, there exists an isomorphism 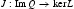.

Lemma 2.1 [10] Let 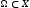 be an open bounded set, *L* be a Fredholm mapping of index zero, and *N* be *L*-compact on 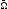. Assume

(i) 
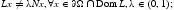

(ii) 
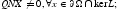

(iii) 
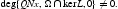

Then *Lx*=*Nx* has at least one solution in 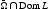.

Note that the second equation and the fourth equation in Equation (7) can be separated from the whole system. Consider the following subsystem of Equation (7):

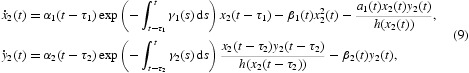

with the initial conditions

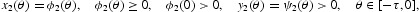

where 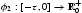, 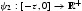 are continuous positive periodic functions. Since we require *x*_2_(0)>0 and *y*_2_(0)>0, each component of the solutions is positive as long as *t*>0 and the solutions are defined.

If 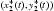 is a positive ω-periodic solution to system (9), then it is not difficult to verify that

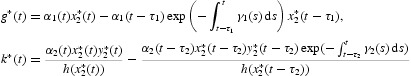

are also ω-periodic by the periodicity of the coefficients of system (7). For system (7), consider the following two linear periodic differential equations:

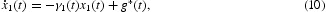

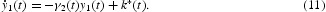

Under the initial condition (8), Equation (10) has a unique solution

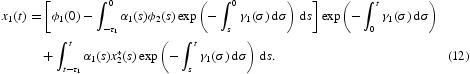

Similarly, Equation (11) also has a unique solution

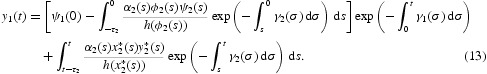

If the following condition holds:

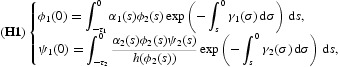

then from Equations (12) and (13), Equation (10) has a unique ω-periodic solution

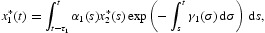

and Equation (11) also has a unique ω-periodic solution

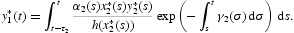

The positivity of 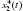, 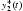 and the coefficients of Equation (7) implies that of 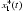 and 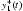. Therefore, in order to prove the existence of multiply positive periodic solutions for system (7), we only need to proof it for system (9).

For simplicity we adopt the following notations throughout this paper:

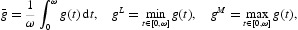

where the function *g*(*t*) is continuous on [0, ω].

Let

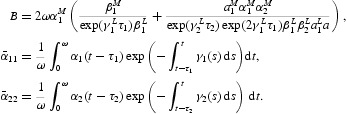



From now on, we assume that

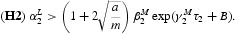

Under assumption (H2), there exist the following six positive numbers:

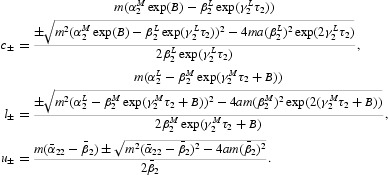

It is not difficult to show that

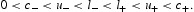



Furthermore, we make the third assumption,

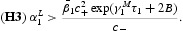



Our result on the existence of multiple periodic solutions to system (7) is stated as the following theorem.

Theorem 2.2 Suppose that the conditions (H1), (H2), and (H3) hold. Then system (7) with the initial condition (8) has at least two positive ω-periodic solutions.

*Proof* From the above analysis, we only need to prove that system (9) has at least two positive ω-periodic solutions. By making the changes of variables

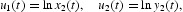

system (9) is rewritten as

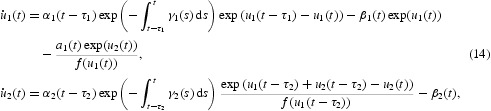

where 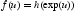. We set

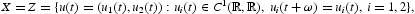

and define the norm of *X* and *Z* as

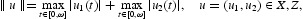

where |·| denotes the Euclidean norm. Then *X* and *Z* are Banach spaces when they are endowed with the usual operations and norm 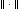. Since 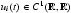, there exist ξ_*i*_ and 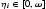, such that

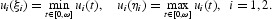



For any 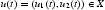, by the periodicity of the coefficients of system (14), we can easily check that both

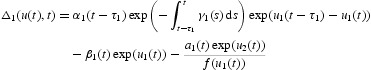

and

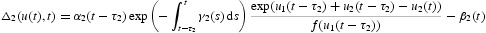

are ω-periodic with respect to *t*. Set

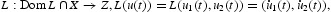

and

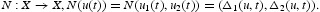

It is not difficult to show that 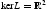 and 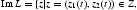 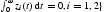 are closed in *Z*. Then 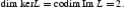 It follows that *L* is a Fredholm mapping of index zero. Define two mappings *P* and *Q* as

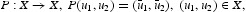

and

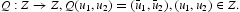

Then *P* and *Q* are continuous projectors such that

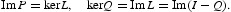

Furthermore, the generalized inverse (of *L*) 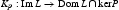 exists and is given by

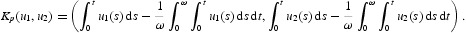

Then 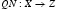 and 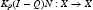 are given, respectively, by

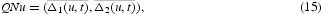

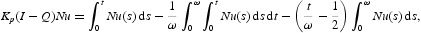

where 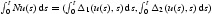. Clearly, *QN* and *K*_*p*_(*I*−*Q*)*N* are continuous. By using the Arzela–Ascoli theorem, it is not difficult to prove that 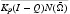 is compact for any open bounded set 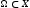. Moreover, 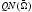 is bounded too. Hence *N* is *L*-compact on 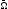 for the open bounded set 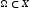. In order to apply Lemma 2.1 to prove the existence of two periodic solutions of system (14), we need to construct two appropriate open bounded subsets in *X*. Corresponding to the operator equation 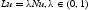, we have

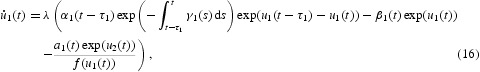

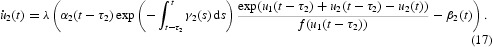

Suppose 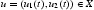 is a solution to Equations (16) and (17) for a certain 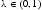. Integrating Equations (16) and (17) over the interval [0, ω], we obtain

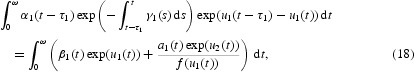

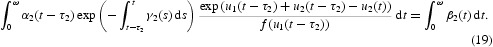

It follows from Equations (17) and (19) that

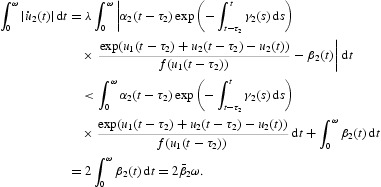

Therefore,

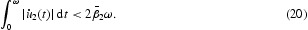

Multiplying Equation (16) by 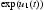 and then integrating it over [0, ω], we obtain

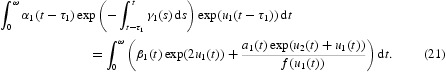

With the inequality

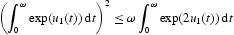

and Equation (21), we get

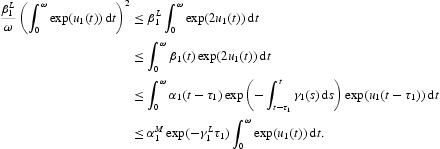

Then we have

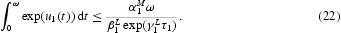

Multiplying Equation (17) by 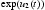, then integrating it over [0, ω], we obtain

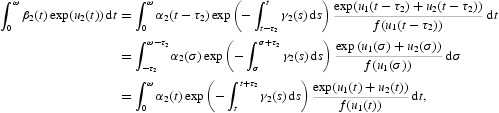

which yields

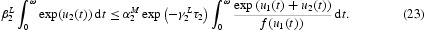

In addition, from Equation (21) we have

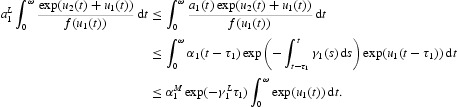

Simply,

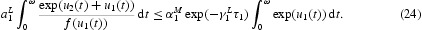

From Equations (23) and (24), we can get

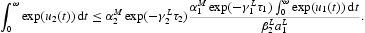

Combining it with Equation (22), we obtain

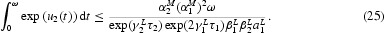

From Equations (16), (18), (22) and (25), we have

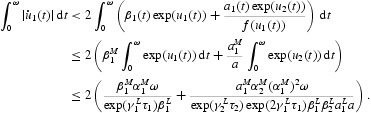

This means

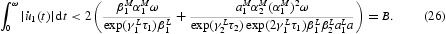

Multiplying Equation (17) by 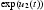 and integrating it over [0, ω], we also obtain

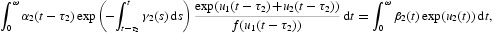

Then,

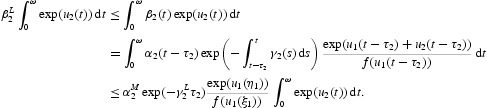

This gives

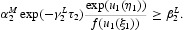

Therefore, we have

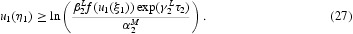

Similarly, we obtain

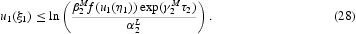

From Equations (26) and (27), it is easy to see that

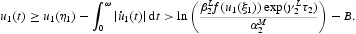

A special case is

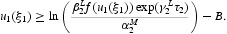

Therefore, we have

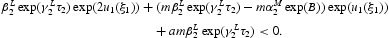

From (H2), it is not difficult to obtain that

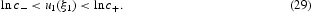

Similarly, by Equations (26) and (28) we get

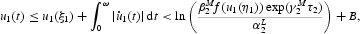

Specially, we have

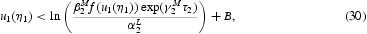

and it follows that

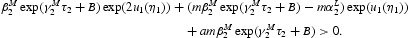

In view of (H2), we have

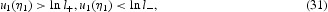

and

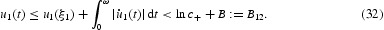

From Equation (21) we can get that

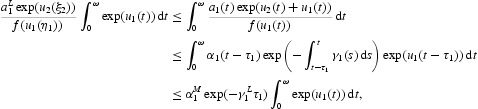

Therefore

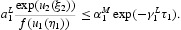

Combining it with Equation (32), we have

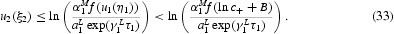

From Equation (21), it also follows that

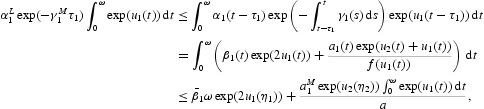

Therefore,

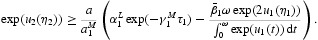

By Equations (29) and (32), we obtain

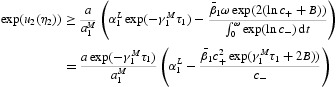

From (H3), we have

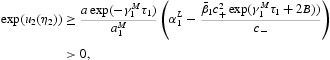

which implies

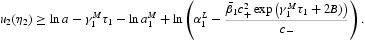

Together with Equation (20), it leads to

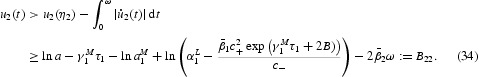

From Equations (20) and (33), we also have

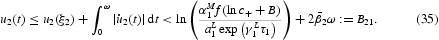

Combining Equation (34) with Equation (35), we obtain

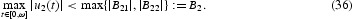

Now we consider *QNu*, where 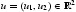. From Equation (15) we have

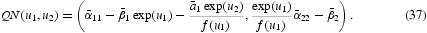

Because of (H2) and (H3), we can show that the equation 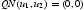 has two distinct solutions

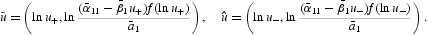

Choose a positive constant *c* such that

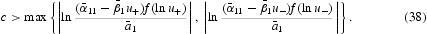

Define

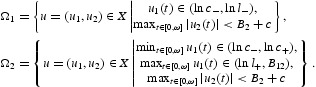

Then, both Ω_1_ and Ω_2_ are bounded open subsets of *X*. It follows from Equations (9) and (38) that 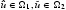. With the help of Equations (9), (29)–(32), (36) and (38), it is easy to see that 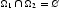, and Ω_*i*_ satisfies the requirement (*i*) in Lemma 2.1 for *i*=1, 2. Moreover, *QNu*≠0 for 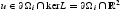. Taking 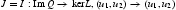, we obtain from Equation (37) that

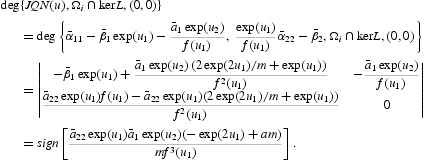



Since *m*, 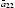, 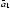, exp(*u*_1_), exp(*u*_2_) and *f*(*u*_1_) are all positive, 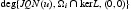 depends on the sign of 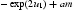. When 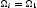, *u*_1_ is equal to ln*u*_−_. Therefore, 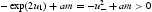. When 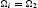, *u*_1_ is equal to ln*u*_+_. Then, 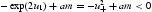. Hence we obtain that 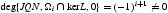. Now we have proved that Ω_*i*_ satisfies all the assumptions in Lemma 2.1. Here, system (14) has at least two ω-periodic solutions 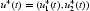 and 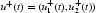 with 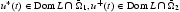. Obviously, u*(*t*) and *u* ^+^(*t*) are different. Let 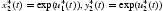 and 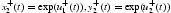. Then 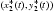 and 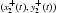 are two different positive ω-periodic solutions to system (9). This completes the proof of Theorem 2.2.

## 3. Example

In system (7), let 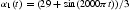, 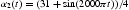, 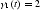, 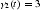, 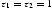, 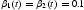, *m*=*a*=1. We have 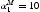, 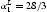, 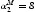, 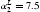, 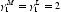, 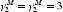, 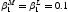, and 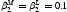.

Taking the initial condition

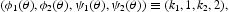

where

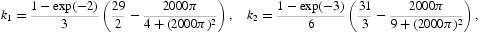

it is easy to verify the assumption (H1) holds.

By computation, we have *c*_−_=0.26, *c*_+_=2.86, *u*_−_=0.41, *u*_+_=2.45, *l*_−_=0.63,*l*_+_=1.58, *B*=0.15. Then we can verify the following two inequalities:

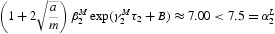

and

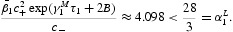

The above inequalities show that assumptions (H2) and (H3) hold. Thus, by Theorem 2.2, system (7) has at least two different positive periodic solutions.

## 4. Conclusion

In this paper, we study the existence of multiple positive periodic solutions to system (7), in which the coefficients are periodic, the predator functional response is non-monotonic, predator and prey species are all divided into immature individuals and mature individuals. By using Mawhin's continuation theorem of coincidence degree theory, we have proved that there exist at least two positive periodic solutions to system (7) under the assumptions (H1),(H2) and (H3). From (H2) and (H3), we know that all parameters of system (7) have effects on the existence of positive periodic solutions and the period ω of the coefficients is an important influence factor on the existence of positive periodic solutions. We found that, when the period ω enlarges, for the existence of periodic solutions to system (7), the infimums of birth rates of prey and the conversion of nutrients into the reproduction rate of mature predator must be increased. In other words, to shorten the period of the environmental change can increase the possibility of the existence of multiple periodic solutions.
